# The Infinity-Lock System for Chronic Grade III AC Joint Dislocation: A Novel Technique, Rehabilitation Protocol and Short Term Results

**DOI:** 10.3390/jcm9082519

**Published:** 2020-08-05

**Authors:** Alfonso Maria Romano, Pasquale Casillo, Monica De Simone, Guglielmo Nastrucci, Donatella Risorto, Massimiliano Susanna, Angelo Di Giunta, Francesco Ascione

**Affiliations:** 1 Orthopedics and Sport Medicine Unit, Campolongo Hospital, 84025 Salerno, Italy; alfonso.maria.romano@gmail.com (A.M.R.); pasquale.casillo87@gmail.com (P.C.); monica.desimone86@gmail.com (M.D.S.); guglielmonastrucci@hotmail.it (G.N.); 2 Department of Orthopaedic and Trauma Surgery, Ospedale Buon Consiglio Fatebenefratelli, 80123 Napoli, Italy; opnisa@tin.it; 3 Orthopedic and Traumatology Unit, San Donà di Piave Hospital, 30027 Venezia, Italy; massimiliano.susanna@gmail.com; 4 Orthopaedic Division of Policlinico ‘G.B. Morgagni’, 95100 Catania, Italy; adigiunta@yahoo.com

**Keywords:** acromioclavicular dislocation, chronic dislocation, infinity lock button system, rehabilitation, surgical technique, clinical results, synthetic ligament

## Abstract

Background: the choice of treatment of chronic grade III acromioclavicular (AC) joint dislocation is controversial. Several surgical techniques have been described in the literature, responding differently to nonoperative treatment. The aim of this study is to describe a modified technique of stabilizing an AC joint dislocation with the new Infinity-Lock Button System, in order to demonstrate that it is effective in optimizing outcomes and decreasing complications. Methods: this is a retrospective study of 15 patients who underwent surgical stabilization of the AC joint dislocation between 2018 and 2019, through modified surgical technique using the Infinity-Lock Button System. Active range of motion (ROM), Specific Acromio Clavicular Score (SACS) and Constant Score (CS) were evaluated preoperatively and postoperatively at last 18 months follow up. Patients rated their outcomes as very good, good, satisfactory, or unsatisfactory. Results: a total of twelve patients rated their outcome as very good and three as good; no patients were dissatisfied with surgery. The mean Constant Score increased from 38 points preoperatively to 95 postoperatively, the average SACS score decreased from 52 points preoperatively to 10 postoperatively, both significantly. No complications were detected. Conclusion: the described technique is effective for treatment of chronic grade III AC joint dislocation, resulting in elevated satisfaction ratings and predictable outcomes. Nevertheless, further longer term follow-up studies are required.

## 1. Introduction

Dislocation of the acromioclavicular (AC) joint is common, accounting for 9% of injuries in the shoulder region with an incidence of 8.9/100,000 per year. After direct trauma to the shoulder it is the most frequent injury seen in the adducted arm and is common among athletes participating in contact sports (such as rugby, wrestling, and hockey) [1,2,3,4]. Force applied to the lateral aspect of the shoulder leads to inferior, medial displacement of the scapula and clavicle, resulting in complete disruption of AC and coracoclavicular (CC) ligaments, and even the muscular attachments of the deltoid and trapezius. 

There is a consensus that minor dislocations, such as type I and type II according to Rockwood, are best treated nonoperatively [5,6]; type IV through to type VI, however, are best managed through surgical reconstruction [7].

Conversely, there is a paucity of randomized controlled trials and prospective comparative studies regarding the treatment of grade III AC joint dislocation and decision making is often based on the individual’s occupation and sporting activity. However, approximately 17% to 28% of patients receiving nonoperative treatment have reported disability with pain, weakness, fatigue, impingement, and AC instability [8].

Surgery is often recommended in patients at increased risk of failure through nonoperative management, such as manual workers, athletes or soldiers who frequently engage in overhead movements [7,9].

A large variety of operative treatment options exist for both acute and chronic AC joint instabilities, summarized in four groups (Table 1): (a) nonbiological fixation between the coracoid and clavicle, including suture loops and synthetic ligaments (polydioxanone (PDS), the Gore-Tex, Dacron, carbon fiber and Mersilene tape [5,10,11,12], the TightRope [13], the Lockdown [14], the Surgilig [15], the ligament augmentation and reconstruction system (LARS) [16,17]); (b) biological reconstruction of the CC ligaments, including allograft or autograft tendon reconstruction (hamstring or palmaris longus autograft) [18,19]; (c) ligament and/or tendon transfer, such as the Weaver–Dunn and Dewar procedures [20]; and (d) fixation with Kirschner wires (Phemister technique) [21], a hook plate [22], or other extra-articular techniques (Bosworth screw fixation [23]).

**Figure 1:**
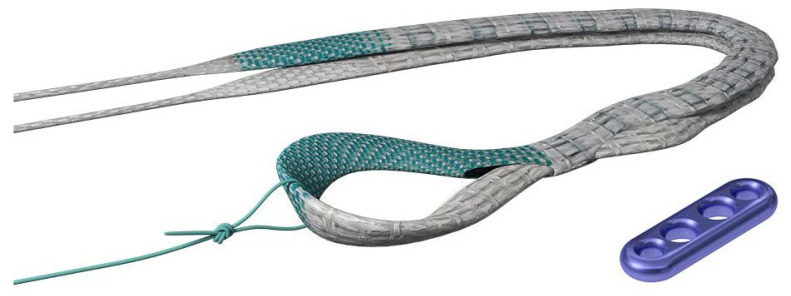
The Infinity-Lock Button System (Neoligaments, Leeds, UK).

More recently, the availability of synthetic ligaments, such as the Infinity-Lock Button System (Neoligaments, Leeds, UK), have provided an alternative to the traditional Weaver–Dunn procedure [14,16,17,24,25,26,27,28]. This comprises a permanent implantable Tube-Tape with integral eyelet which is looped around the coracoid, together with a titanium button for clavicle attachment, maintaining a reduced anatomical (vertical) position during implantation of the device (Figure 1), providing a mini-open technique which is non-harvesting, simple and reproducible for the stabilization of a dislocated AC joint, due to CC ligament disruption. This technique is used for Grade III, IV, VI AC separations. 

Our hypothesis was that the described modified technique of stabilizing AC joint in grade III dislocation with the new Infinity-Lock Button System is effective in optimizing outcomes and decreasing complications, and we aim to report the clinical short-term results of this case series. A further objective is to standardize a rehabilitation protocol.

## 2. Material and Methods

The Institutional Review Board of the ethical committee of the Campolongo Hospital read the study project entitled: “The Infinity-Lock System for Chronic Grade III AC Joint Dislocation: Modified Technique, Rehabilitation Protocol and Short Term Results”. This project does not infringe the Italian ethical rules and the privacy of the patients.

The present study comprises a retrospective study of patients who underwent surgical stabilization of AC joint dislocation between 2018 and 2019, through the use of the Infinity-Lock Button System, which employs a woven Tube-Tape, 7 mm wide by 240 mm long with an integral eyelet. Each limb tapers into a 140 mm long cord. This continuous structure is made from polyethylene terephthalate (polyester). The Tube-Tape is attached to a Button made from implant grade titanium alloy.

The study inclusion criteria were: (1) primary AC dislocation of grade III, according to Rockwood; (2) time elapsed since trauma ≥6 weeks; and (3) patients under 60 years old. 

**Scheme 1:**
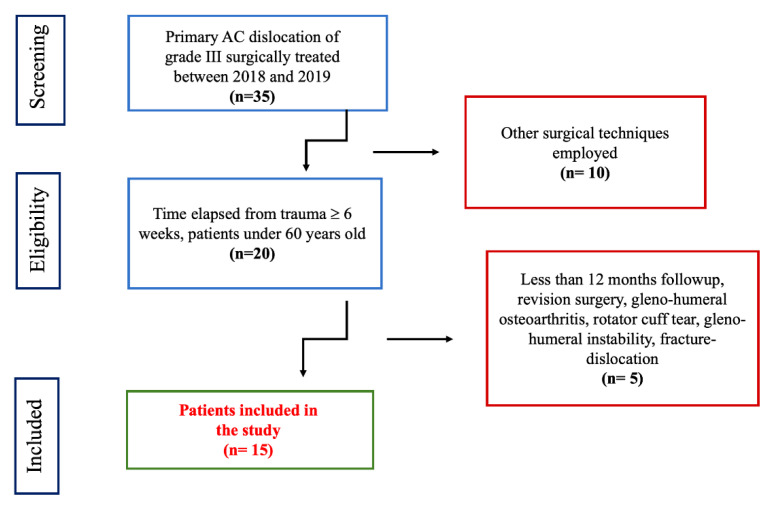
Patients recruiting process.

Exclusion criteria were: (1) revision surgery, (2) gleno-humeral osteoarthritis, (3) rotator cuff tear, (4) gleno-humeral instability, (5) fracture-dislocation (Scheme 1). 

All patients gave their agreement prior to enrolment and the study was approved by the local hospital institutional review board. 

All patients were evaluated pre-operatively, at 2 weeks, 4 weeks, 8 weeks, 3 months, 6 months and every year postoperatively. 

Active range of motion (ROM) was measured pre-operatively and postoperatively, with active forward flexion (FF), external rotation with the elbow at the side (ER1) and 90° abduction (ER2), and internal rotation with level of the thumb on the spine (IR1) and 90° abduction (IR2).

Objective functional results were evaluated preoperatively and postoperatively at last follow up, through Specific Acromio Clavicular Score (SACS) [29] and Constant Score (CS) [30], ranging from 0% to 100%. 

The patients were asked by the examiner to rate their outcome as very good, good, satisfactory, or unsatisfactory. Return to work/sport was also recorded. 

Standardized radiographic films, including a true anterior–posterior view at three different rotations of the arm (internal, external and neutral), and the scapular Y-view were obtained pre- and post-operatively at 4 weeks, 3 months, and on last follow-up. 

The clinical post-operative evaluation was performed by two independent examiners who were not involved in the surgery (F.A. and P.C.).

### 2.1. Surgical Technique

All procedures were performed by two senior shoulder surgeons (A.M.R. and A.D.G.), with patients in the beach-chair position, under an interscalene nerve block with or without intravenous sedation. The operated arm was draped freely, without any traction. Patients were placed on prophylactic antibiotics prior to surgery, to minimize the risk of latent infections developing at the implant site. The present study describes a deviation from approved original Infinity-Lock Button System (Neoligaments, Leeds, UK) technique.

**Figure 2:**
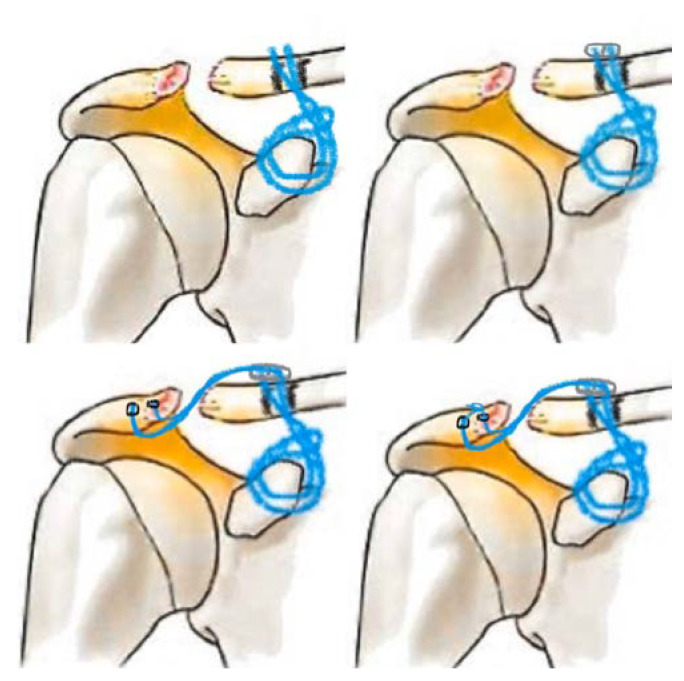
Surgical procedure outlined in concise drawings.

The surgical procedure is clearly outlined in Figure 2.

Through a vertical 5 cm skin incision starting at the level of the clavicle and slightly medial to the tip of the coracoid, the fascia is incised vertically, the deltoid minimally detached and the periosteum is then divided over the posterior clavicle laterally as far as the AC joint.

Carrying out a sub-periosteal dissection and creating an “L” shaped flap in the apex of the flap as a stay suture and to aid retraction, a self-retaining retractor is applied to aid access to the coracoid process and to clear soft tissues from around the coracoid.

The distal clavicle oblique 45°–60° osteotomy is performed by means of a saw, 1 cm from the joint, in a medial to lateral direction starting superiorly. 

A coracoid passer (the Neoligaments CC-Hook) is first inserted under the neck of the coracoid from medial to lateral, taking care to avoid potential injury to the medial structures and musculo-cutaneous nerve, thus capturing the lead suture of the Tube-Tape with the coracoid passer. 

The coracoid passer below the coracoid is then withdrawn medially, simultaneously pulling the lead suture around the bone, and both limbs of the Tube-Tape are passed through the loop, lassoing the coracoid and tightening the noose around the coracoid.

Before identifying the tunnel location, dislocation reduction is achieved by pushing downward on the clavicle while simultaneously pushing up on the elbow to support the arm. 

After identifying a point 1.5 cm to 2.5 cm from the lateral end of resected clavicle (ligament attachment), a 2 mm guidewire is drilled perpendicularly through the middle of the clavicle at this point; a 4 mm cannulated drill is employed to create the final bone tunnel in the clavicle. A retractor is placed inferiorly to the clavicle to prevent damage to surrounding tissues when the drill bit breaks through the cortex.

In order to minimize the distance from the exit of the bone tunnel to the noose, the angle of the fully tightened loop around the coracoid is adjusted; the nitinol wire from the CC-Hook is used to pass the limbs of the Tube-Tape through the bone tunnel one at a time and sutured, via the central holes of the Infinity-Lock Button, should it be necessary.

The Button is pushed down until it locates against the clavicle and a half-knot is tied over the top of the Button. This half-knot helps maintain reduction during the procedure; a single throw has sufficient strength and further knots may cause tissue irritation. 

**Figure 3:**
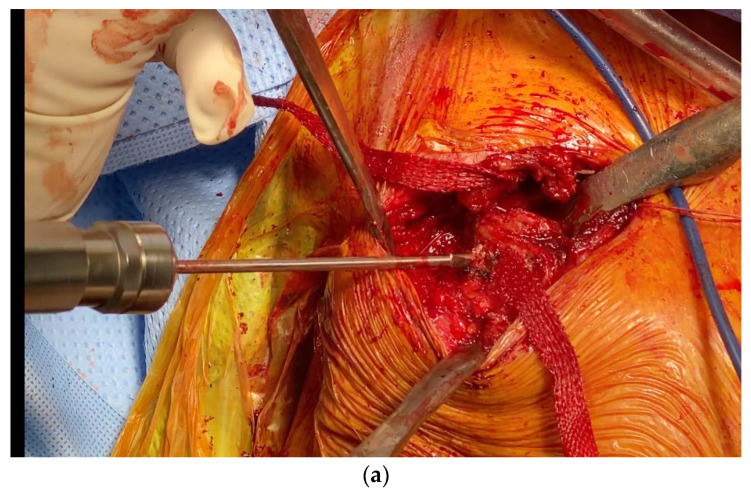

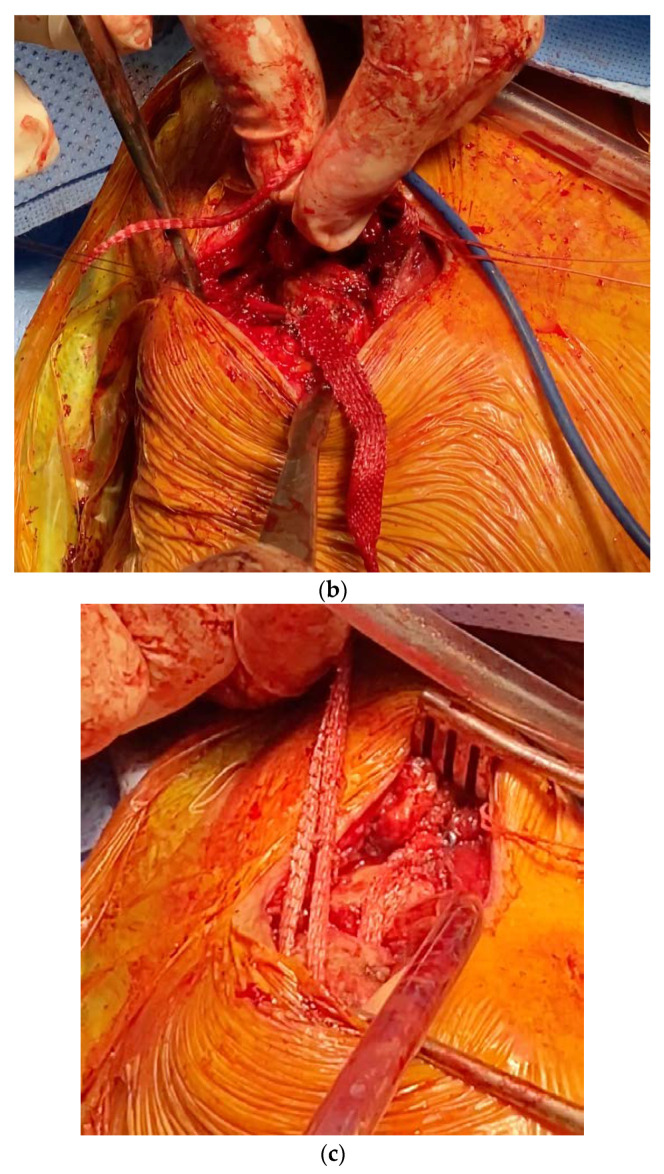
The modification of technique, with final phases. (**a**) a guidewire is drilled twice, perpendicularly, through the acromial process, (**b**) the CC-Hook is used to pass the limbs of the Tube-Tape through the two bone tunnels, running from the inferior to the superior surface of acromion, (**c**) final construction.

In addition, a 2 mm guidewire is drilled twice, perpendicularly, through the acromial process at 1 to 1.5 cm from the AC joint, first anteriorly and then posteriorly (Figure 3a). Again, the nitinol wire from the CC-Hook is used to pass the limbs of the Tube-Tape through the two bone tunnels, running from the inferior to the superior surface of acromion (Figure 3b). This modification to the original technique is thought to obtain increased AC joint stabilization and a more resistant construction, opposing both superior–inferior forces (coracoid–clavicle) and anterior–posterior as well as medial–lateral forces (acromion–clavicle) (Figure 3c).

Final repair is checked physiologically and that it is not affecting range of motion. When satisfied, a surgeon’s knot secures the stabilization on the superior or anterior surface of the acromion.

**Figure 4:**
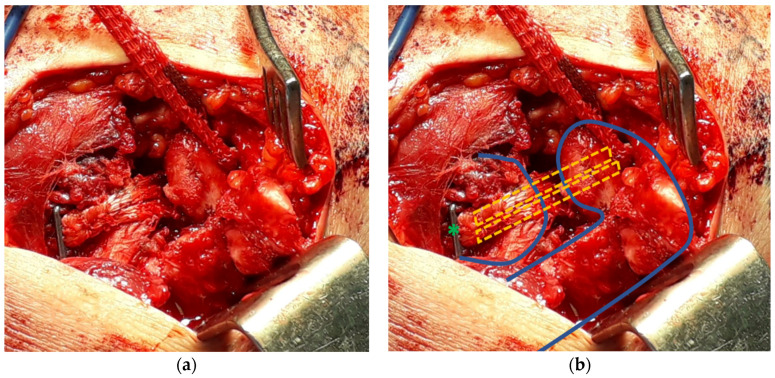
The final surgical result. (**a**) The final surgical result., (**b**)the final construction (blue lines define acromion and clavicle, yellow dotted lines the synthetic ligament under the acromio-clavicular joint and the green asterisk (*) indicates the Infinity Lock titanium button.

Finally, soft tissues are repaired by re-attaching the “L” shaped flap while tensioning the superior acromioclavicular ligament during the repair and ensuring the severed ends of the Tube-Tape are well embedded in tissue (Figure 4a,b). 

### 2.2. Rehabilitation

A personalized rehabilitation protocol was employed after surgery [31]. All patients were made to use a sling immobilizer postoperatively for 4 weeks. Gentle pendulum and Codman’s exercises were introduced on postoperative day 1. 

At 4 weeks postoperatively, physiotherapy with passive motion and cuff isometrics was started. A resistive exercise program was introduced at 8 weeks postoperatively.

Patients were generally allowed to return to manual work at 2 to 4 months, depending on the level of rehabilitation. Contact sports were not allowed for 6 months postoperatively. 

The range of motion must be such as to avoid stress on the joint, therefore internal rotation movements behind the back, adduction and elevation to extreme degrees were not permitted.

Once complete passive ROM was achieved, isometric exercises of rotator cuff and stabilizers of the scapula muscles were introduced; isotonic and reinforcement exercises were gradually added for the supraspinatus and deltoid and eccentric exercises of the rotator cuff.

Therefore, in the first phase, closed kinetic chain exercises were recommended because they minimize the effort of rotator cuff muscles in supporting the arm. These easy and safe to perform exercises, that focus on reinforcing the scapula stabilizers since the fundamental objective in this phase of rehabilitation is to obtain good scapular stability; for example, scapular retraction exercises or the scapular clock are useful to this end. When the patient was able to maintain the anterior elevation position without pain or weakness, it was possible to introduce isotonic exercises and exercises with an open kinetic chain, also combining movements of the lower limbs and body with shoulder exercises, to improve the normal upper limb movement patterns.

Finally, more complex strengthening exercises of the scapula stabilizers are included in the rehabilitation program, such as the strengthening exercises of the middle and lower fibers of the trapezius with the patient in a prone position, with weights added, gradually.

The rehabilitation program ends with exercises in the field of throwing and grasping objects on different surfaces, education in movement and prevention of recurrence. 

### 2.3. Statistical Analysis

The statistical analysis was performed using the Social Science Statistics collaborative website (http://www.socscistatistics.com). The Student’s *t*-test for paired data was used to compare differences between preoperative and final follow-up data; the Fisher test or the Chi-square test was used to identify the relationships between variables. Significance was set at *p* < 0.05.

## 3. Results

The group comprised 15 male patients affected by a grade III AC dislocation. The dominant arm was affected in 10 patients. Mean age at the time of surgery was 32 years (range, 24 to 45 years). Minimum follow up was 12 months and the mean duration of follow-up was 18 months (range, 12 to 24 months). The mean time between trauma and AC joint stabilization was 8 weeks (range, 6 to 12 weeks). 

A total of twelve patients rated their outcome as very good and three as good; no patients were dissatisfied with surgery. 

The mean Constant score increased from 38 (range, 30 to 63) points preoperatively to 95 (84 to 100) postoperatively. The average SACS score decreased from 52 (range, 20 to 70) points preoperatively to 10 (0 to 30) postoperatively. The mean forward flexion of the shoulder increased from 70° (range, 45° to 120°) preoperatively to 162° (100° to 170°) postoperatively. Clinical outcomes are shown in Table 2. The average Constant score, SACS score, forward flexion and external rotation all improved significantly.

**Figure 5:**
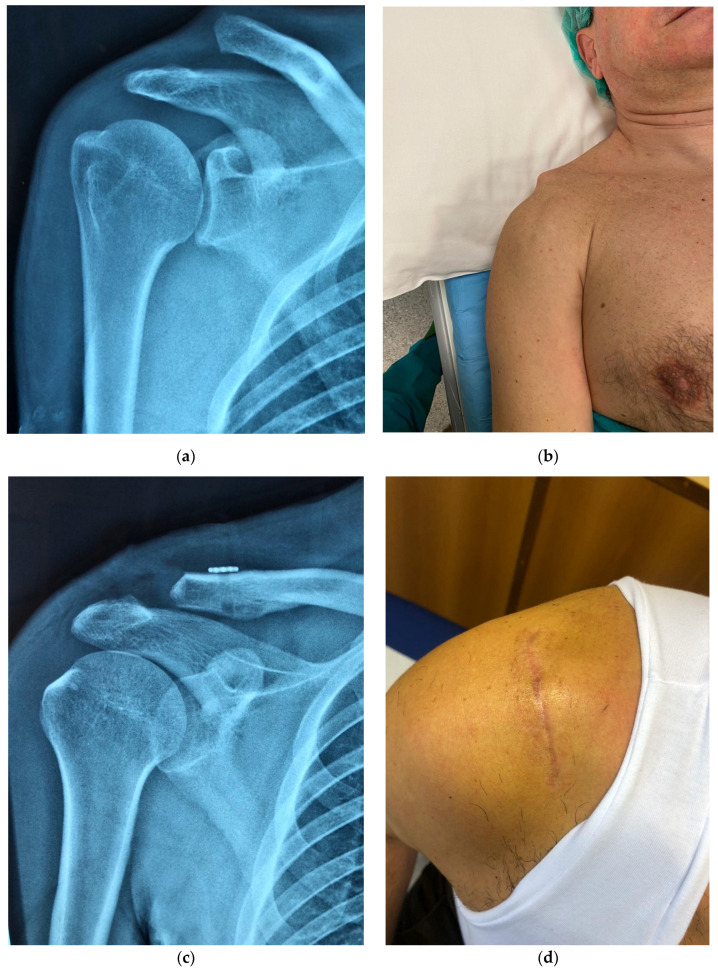
A clinical and radiological pre- and post-operative example. (**a**) preoperative X-ray, (**b**) clinical visualization of dislocation, (**c**) postoperative 3 months X-ray, (**d**) post-operative 3 months consultation.

No intraoperative (bone fracture, implant breakage) and postoperative complications were recorded (wound infection or clavicular/coracoid process fracture or bone remodeling, required implant removal for irritation, recurrent dislocations, impingement or bursitis) (Figure 5). No clinical or radiographic loss of AC joint reduction were found at last follow up. 

## 4. Discussion 

A variety of surgical techniques have been described in the literature for the treatment of grade III injuries which appear heterogeneous, and hence respond differently to nonoperative treatment; as a result, there is controversy regarding the treatment of these injuries [6,32] (Table 1).

In the surgical management of AC joint injuries, the timing of surgical intervention is a clinically relevant factor. Previous studies have shown the treatment of chronic AC dislocations 6 or more weeks after the accident [33] results in less favorable outcomes and higher complication rates compared to acute repair, with an about 78% compared to 90% success rate of chronic and acute dislocation repair, respectively [15,17,34,35,36,37].

Although a systematic review reported that more than 150 variations have been described in the treatment of symptomatic AC joint dislocations, [37] to date, no reconstruction technique can duplicate the stability and physiology of a native, intact AC joint complex [38].

It has been demonstrated that, anatomically, coracoclavicular (CC) ligaments are a major contributing factor to the stability of the AC joint. Therefore, the CC interval should be adequately supported until biological healing of the soft tissue around the CC ligaments occurs [39]. Clinical evidence, although limited, suggests that anatomical ligament reconstruction with autograft or certain synthetic grafts may have better outcomes than non-anatomical transfer of the coracoacromial ligament [40], as is discussed in the present study. It has been suggested that this is due to better restoration of the horizontal and vertical stability of the joint, the rationale behind the modified surgical technique we described above.

All surgical treatments evaluated in the literature reported improved subjective patient-reported outcomes and low unplanned reoperation rates [5,6,7,8,15,20,36].

Tauber et al. [27] prospectively compared AC joint stabilization via free semitendinosus autograft vs. a modified Weaver–Dunn procedure, with significantly better results both clinically and radiographically in the free tendon graft group. In the other study, Fauci et al. [16] prospectively compared allograft tendon vs. synthetic ligament reconstruction (LARS) in 40 patients. In that study, the allograft tendon group achieved significantly better clinical scores than the synthetic ligament group at both 1 and 4 years. In a study by Kumar et al. [41], open AC joint reconstruction with a modified Weaver–Dunn technique was compared to Surgilig CC ligament reconstruction, the polyester ligament showing significantly higher postoperative scores, as well as an earlier return to work and sports. Fraschini et al. [17] published a retrospective comparative study with three different treatment groups and 30 patients per group: (1) conservative therapy, (2) an AC joint reconstruction technique with a Dacron prosthesis, and (3) LARS reconstruction. Both the Dacron and LARS groups had better clinical results; the Dacron group, however, had a high complication rate (43%). 

Reconstruction of the CC ligament using synthetic ligaments, such as the Lockdown, the Surgilig, the LARS, as opposed to rigid fixation methods, has proven to be an effective technique for the management of AC joint dislocations in biomechanical and clinical studies [17,42], and preservation of the native coracoacromial ligament, as is well acknowledged in the literature [43,44]. Malahias et al. [15] reported in a systematic review that the failure rate of the Surgilig implant was very low (3.5%), while patient satisfaction was high (88.3%). However, the quality of most of these studies was low. The present study reports significant improvement in all the outcomes at final follow up.

Moreover, serious concerns still exist regarding previous fixation methods, and using screws, plates, and K-wires has been associated with hardware complications (implant breakage or migration, ligament failure and recurrent dislocations, incomplete reduction, foreign body reaction, bony erosion, fractures) [34,37,39,42].

Suture button systems and free tendon grafts necessitate drilling bone tunnels in the clavicle and, at times, in the coracoid, thereby increasing the risk of intraoperative and postoperative fractures [45,46,47]. The Infinity Lock Button system, through the loop fixation around the coracoid, eliminates the need for a bone tunnel and the single 4 mm diameter clavicle tunnel reduces the risk of bone fracture. Furthermore, a non-absorbable polyester scaffold construction allows provides fixation during the healing process [28,48] and eliminating the need for tissue graft and its associated morbidity (autograft), or additional cost (allograft). No perioperative complications were recorded in our series.

The overall complication rate lies between 6% and 30% for all techniques [5,20,38,46].

In a recent review [20], the ligament and/or tendon transfer group showed a higher complication rate (18%), but the lowest rate of secondary loss of reduction, at 5.3%. Tendon grafts are prone to harvest site complications, such as hypoesthesia, pain or wound related problems (5%), as well as infections (with a similar percentage in the Weaver–Dunn procedure/conjoined tendon transfer (7.4%)), and additional surgical time.

In another systematic review, Moatshe et al. [5] reported a complication rate of 26.3% in patients treated with a hook plate or K-wires in both acute and chronic stabilizations, with suspensory devices and synthetic ligament techniques showing the lowest rates of complication at 6.2% and 4.4%, respectively; unplanned reoperation rates were 1.2%, 2.8%, 0.9%, 5.4%, and 2.6% in free tendon graft, suspensory devices, synthetic ligament devices, modified Weaver–Dunn, and hook plate/K-wires techniques, respectively. Previous studies have reported complication rates for these procedures to be as high as 30% [38], comprising loss of reduction (29%), clavicle fracture (18%), infection (6%), and hardware-related issues (4%) whereas free graft reconstruction provided the highest subjective scores and fewest complications. 

Woodmass et al. [46] conducted a systematic review of complications after arthroscopic fixation of the AC joint. They showed low overall rates of serious complications, such as infection requiring further surgery or neurovascular compromise (3.8% only superficial infection), while the rate of fracture and loss of reduction remained a concern. Clinical and radiographic failure rates of 50% or more (some after bone tunnel widening) were reported in chronic dislocations, whether using an anatomic or nonanatomic graft. The safest and most predictable results for AC joint reconstruction were obtained using TightRope/Endobutton techniques in patients with acute separations, but with hardware migration into the clavicle, the coracoid, or both, as high as 89%.

Perioperative coracoid and/or clavicle fracture was classified significant at 5.3%, but many studies included in this review demonstrated a fracture rate of up to 20%.

Precautions should be taken to avoid these fractures which are most likely related to bone tunnel location, size, and proximity to the distal clavicle or other bone tunnels. Extreme care should be taken intraoperatively to ensure accurate placement of bony tunnels through the center of the bone on a single pass, and to maximize the distance between other bony tunnels and the terminal bone end. 

In the technique presented, resection of the distal clavicle was employed. This can be performed as an isolated procedure, or as part of a more complex procedure for treatment of AC joint injuries. Distal clavicle excision generally results in good to excellent outcomes and has been used in several studies as an additional procedure for the reduction of the dislocated clavicle [26,49], but no definite conclusions can be drawn regarding the role distal clavicle excision might play in AC joint stabilization postoperatively.

### Limitations

Shortcomings inherent to a non-randomized and retrospective study were the major limitations. In addition, the retrospective design may have resulted in under-reporting of complications and there is inadequate follow-up to assess long-term clinical and radiographic outcomes as the rate of complication or need for reoperation surgeries may increase over time.

However, the present study has several strengths: it was conducted over a short period and a limited indication, using clinical and radiological assessments, surgical techniques and rehabilitation protocols which were standardized for both surgeons.

Despite the relatively small number of cases in the current study, the results are quite promising.

The cohort of patients is small but is consistent with other studies.

## 5. Conclusions

In conclusion, the technique described is effective for the treatment of chronic grade III AC joint dislocations, resulting in high rates of satisfaction and predictable outcomes. Results are promising, but further and longer follow-up studies are required.

## Figures and Tables

**Table 1:** Main Techniques employed in acromioclavicular (AC) stabilization.

Technique	Advantages	Disadvantages
Weaver-Dunn/ Dewar	Widely used in literature	Sacrifice of CA ligament/conjoint tendon, lower strength of construct, may require augmentation
Bosworth	Low cost, readily available	Bicortical and bi-bone fixation, risk of fracture/screw loosening or breakage, may require removal
Hook Plate/ K wires	Strong construct	Plate impingement, skin and infections problems, second operation required
Tendon grafts	Good outcomes, biological construct, no sacrifice of CA ligament	Donor site related problems, infections, additional costs for allograft, additional surgical time
Synthetic ligaments	Biomechanically proven, non-harvesting technique, no sacrifice of CA ligament, enable soft tissue ingrowth, good tensile strength, low complications rate	Possible soft tissue irritation/reaction, fixation screw may require removal, bone fracture risk
Suture loops/ suspensory systems	Biomechanically proven, non-harvesting technique, loop around coracoid, strong fixation, no sacrifice of CA ligament	Bone fractures, loss of reduction, bony erosion, implant migration
Infinity Lock System	non-harvesting technique, single 4mm clavicle tunnel, loop around coracoid, no sacrifice of CA ligament, good tensile strength, titanium button on clavicle maintains reduction, low complications rate	Possible soft tissue irritation/reaction

**Table 2:** Clinical results.

	PRE	POST	*p*
FF	70 ± 28	162 ± 8	<0.05
ER1	15 ± 5	35 ± 10	<0.05
ER2	46 ± 9	84 ± 5	<0.05
IR1 (out of 10), points	5 ± 3	9 ± 1	NS
IR2	38 ± 9	88 ± 1	<0.05
SACS	52 ± 15.7	10 ± 8.2	<0.00001
CS	38 ± 15	95 ± 3	<0.00001

PRE, preoperative; POST, postoperative; FF, forward flexion; ER1, External rotation with elbow at side; ER2, external rotation at 90° of abduction; IR1, active internal rotation hand level; IR2, internal rotation at 90° of abduction; SACS, Specific Acromio Clavicular Score; CS, Constant Score. Values given are Mean ± Standard Deviation.
